# Minimal Residual Disease and Survival Outcomes in Patients with Mantle Cell Lymphoma: a systematic review and meta-analysis

**DOI:** 10.7150/jca.51959

**Published:** 2021-01-01

**Authors:** Yu Zhou, Haizhu Chen, Yunxia Tao, Qiaofeng Zhong, Yuankai Shi

**Affiliations:** Department of Medical Oncology, National Cancer Center/National Clinical Research Center for Cancer/Cancer Hospital, Chinese Academy of Medical Sciences & Peking Union Medical College, Beijing Key Laboratory of Clinical Study on Anticancer Molecular Targeted Drugs, Beijing, 100021, China.

**Keywords:** minimal residual disease, mantle cell lymphoma, clinical outcome, prognosis, meta-analysis

## Abstract

**Background:** Minimal residual disease (MRD) has shown the prognostic value in mantle cell lymphoma (MCL). To quantify the relationships between progression free survival (PFS) and overall survival (OS) with MRD status in MCL, we conducted this meta-analysis.

**Methods:** We searched databases including Pubmed, Embase, Web of Science and the Cochrane Library up to July 15^th^, 2020. Data of patients' characteristics, MRD assessment and survival outcomes were extracted and analyzed.

**Results:** Ten articles were included. For the impact of post-induction MRD status on survival outcomes, MRD positive status was associated with worse PFS (HR=1.44; 95%CI 1.27-1.62; *P*<0.00001) and OS (HR=1.30; 95%CI 1.03-1.64; *P*=0.03) compared with MRD negative status. Regarding the impact of post-consolidation MRD status on survival outcomes, MRD positivity predicted shorter PFS (HR=1.84; 95%CI 1.49-2.26; *P*<0.00001) and OS (HR=2.38; 95%CI 1.85-3.06; *P*<0.00001) than MRD negativity.

**Conclusions:** This study indicated that MRD positivity after induction and consolidation treatments was associated with worse PFS and OS for MCL. MRD-based treatment strategies should be further explored in clinical trials and real-world practice.

## Introduction

Mantle cell lymphoma (MCL) is an uncommon subtype of non-Hodgkin lymphoma (NHL), comprising about 3% of NHL 1. Most patients are diagnosed with advanced stage disease, considered as incurable by conventional chemotherapies 2. In recent years, the application of high-dose chemotherapies, chemoimmunotherapies and non-chemotherapeutic agents such as frontline induction treatment, as well as autologous stem cell transplantation (ASCT) consolidation, and rituximab maintenance after ASCT has improved long term survival of MCL 3-8. However, although these therapeutic strategies bring survival benefits, most patients will still relapse eventually. Also, the toxicities and economic burden from consolidation and maintenance treatment can still be the problems 9, 10. Therefore, early assessment of treatment efficacy and response-guided therapy are necessary in MCL management.

Minimal residual disease (MRD) refers to the persistent traceable tumor cells in patient's peripheral blood (PB) or bone marrow (BM) after treatment. Recent years, MRD detection has shown specificity and sensitivity for diagnosis and efficacy assessment in NHL 11. MRD represents the depth of molecular remission and provides evidence for early response of treatment efficacy. Previous studies showed that MRD assessment could predict survival outcomes in MCL 12-16. Also, in some previous clinical trials, surveillance of MRD can monitor response to prior therapy and may inform the need for further consolidation or maintenance therapy in MCL 17, 18. Due to the low incidence of MCL and the complexity of conventional detection techniques, previous single studies regarding MRD and survival outcomes were mostly from relatively small sample size. To further understand the impact of MRD on survival outcomes in newly diagnosed MCL, we conducted this systematic review and meta-analysis.

## Methods

This systematic review and meta-analysis was performed in accordance with the Preferred Reporting Items for Systematic reviews and Meta-Analyses (PRISMA) guidelines^19^ and has been registered on International Prospective Register of Systematic Reviews (PROSPERO) (register ID: CRD42020192171).

### Literature search

Literature search was performed for articles from electronic databases including Pubmed, Embase, Web of Science and the Cochrane Library up to June 15^th^, 2020, using the MeSH terms “neoplasm, residual” and “Lymphoma, Mantle-Cell” and the free text words “Minimal Residual Disease”, “Residual Tumor”, “Residual Cancer”, “MRD”, “Mantle Cell Lymphoma” and “MCL”. The complete search strategies were described in supplementary Table S1.

### Eligibility and study selection

Full-text articles in English of randomized controlled trials or patient cohort studies were included. The main eligible criteria were as following: (1) studies in which patients were with newly diagnosed MCL and received no prior treatment; (2) studies in which the association between MRD and survival outcomes was reported; (3) studies from which the data of interest could be extracted. When the studies reported data from the same patient cohort or the patient cohort was overlapping, the study with most updated and complete data would be included. Outcomes of interest were progression-free survival (PFS) and overall survival (OS). Data were analyzed for PFS and OS grouped by MRD detection time. Two investigators (YZ and HZC) worked independently to assess the eligibility of studies. If there was any disagreement, studies would be re-assessed by the third investigator (YXT).

### Data Extraction and Quality Assessment

Two investigators (YZ and HZC) worked independently to extract data from included studies. The following information was extracted from the publications if available: region of the study, study sample size, MRD source, MRD detection method, MRD detection time, MRD cut-off value, median follow-up time, patients' median age, treatment for induction, treatment for consolidation and treatment for maintenance. When extracting hazard ratio (HR) and 95% confidence interval (CI) for aggregation and comparison, we used the following methods to get information needed: (1) when the publications provided HR and 95% CI, we extracted the direct data; (2) when the publications provided number at risk, observed number (O) of events and the differences (O-Es), or Kaplan-Meier curves of PFS and OS, we regenerated survival results using methodology described by Tierney et al. 20. Engauge Digitizer software (version 11.1) was applied to extract coordinates of points on the curves. Studies were excluded if HR and 95% CI cannot be extracted using above methods.

Newcastle-Ottawa quality assessment scale was applied to assess the selection and comparability and outcomes of study cohorts 21. The total scores ranged from zero to nine points, with a score of lower than five indicating poor quality, five to seven indicating medium quality, and higher than seven representing high quality. Two investigators (YZ and HZC) worked independently to assess quality of the studies.

### Statistical Analyses

The outcomes of interest were PFS and OS. For pooled analysis of studies reporting MRD status and survival outcomes, estimated survival curves were generated using methodology described by Combescure et al. 22. HR and 95% CI were used to conduct a pooled HR and 95% CI for survival outcomes. Random-effects meta-analysis using DerSimonian and Laird method was applied to conduct pooled treatment effects from included studies 23. Cochrane *Q* test and *I^2^* statistic were applied to estimate the heterogeneity among included studies. For *Q* test, *P* value <0.05 indicated significant heterogeneity; for *I^2^* statistics, *I^2^* value >50% suggest substantial heterogeneity 24. Funnel plots, Begg's test and Egger's tests were used to evaluate the publication bias 25, 26. Trim and fill method were applied for testing and adjusting publication bias 27. Sensitivity analysis using random-effects model was performed to detect the potential source of heterogeneity. R software (version 3.6.2, https://www.R-project.org/) was applied for all the data analysis. Two-side *P* value <0.05 was considered as statistically significant.

## Results

### Literature search and study selection

**Figure 1:**
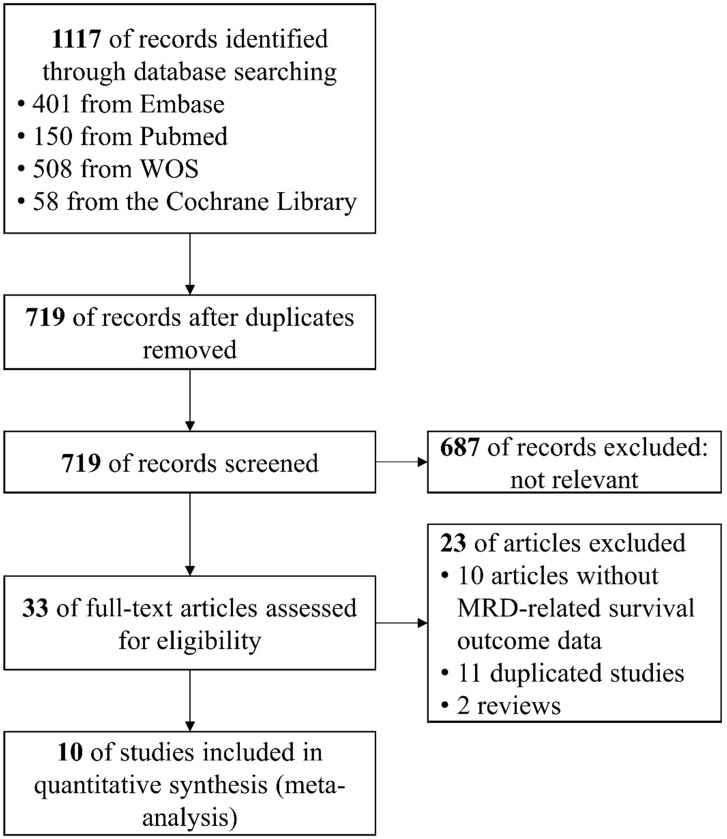
Flow chart of the search and screening process.

In total, 719 records were identified and ultimately ten studies were considered eligible for quantitative meta-analysis 4, 12-16, 28-31. The procedure of study selection was illustrated in Figure 1. Of the included ten studies, ten studies reported MRD-related PFS and six studies reported MRD-related OS. Of note, the study by Pott et al. (2010) 13 aggregated data from two independent cohorts (MCL Younger Study and MCL Elderly Study) and data from MCL Younger Study was updated in the trial by Hermine et al. (2016) 4. Hence, we only extracted data of MCL Elderly Study from the report by Pott et al. (2010).

For the quality of trials, included studies were assessed as low to moderated risk of bias. The results of Newcastle-Ottawa Scale for quality assessment were shown in supplementary Table S2.

### Patients' characteristics

Median age of patients ranged between 55 to 73 years old across the ten studies. The induction therapies were multiple, including chemotherapy and chemoimmunotherapy. Six studies included ASCT-eligible patients 4, 12, 14-16, 29 and in two of the six studies, patients received post-consolidation maintenance, using rituximab or bortezomib 14, 16. Patients did not receive ASCT in the other four studies 13, 28, 30, 31 and in two of the four studies, patients received post-induction maintenance using interferon-α or rituximab 13, 30 (Table 1).

### MRD assessment

As for the timing of MRD detection, in nine studies, MRD was assessed after the completion of induction treatments 4, 13-16, 28-31. Four studies reported MRD status and the related survival outcomes after ASCT or during maintenance 4, 12, 13, 15. The four studies monitored post-consolidation MRD status in similar protocol. In the trial by Pott et al. (2006) 12, post-consolidation MRD was monitored from months 3, 6, 9, and 12 after ASCT. In the trial by Hermine et al. (2016) 4, MRD status was assessed at clinical staging during every 3 months follow-up. In the trial by Kolstad et al. (2017) 15, MRD evaluation was performed at 2-3 months, 6 months post-ASCT and then every 6 months until relapse or 5 years follow-up was completed. In the trial by Pott et al. (2010) 13, MRD status was monitored at 2- to 3-monthly intervals during maintenance treatment. Only one study reported the impact of mid-induction treatment MRD status on survival outcomes 30. Due to this reason, we did not perform meta-analysis for mid-induction MRD status.

Peripheral blood and bone marrow were obtained for MRD assessment. One study adopted both polymerase chain reaction (PCR) and flow cytometry (FC) as detection method 13 while the other nine studies adopted PCR as detection method. All the included studies explored the association between survival outcomes and MRD with a minimum sensitivity of 10^-4^ (Table 1).

### Meta-analysis of post-induction MRD status

**Figure 2:**
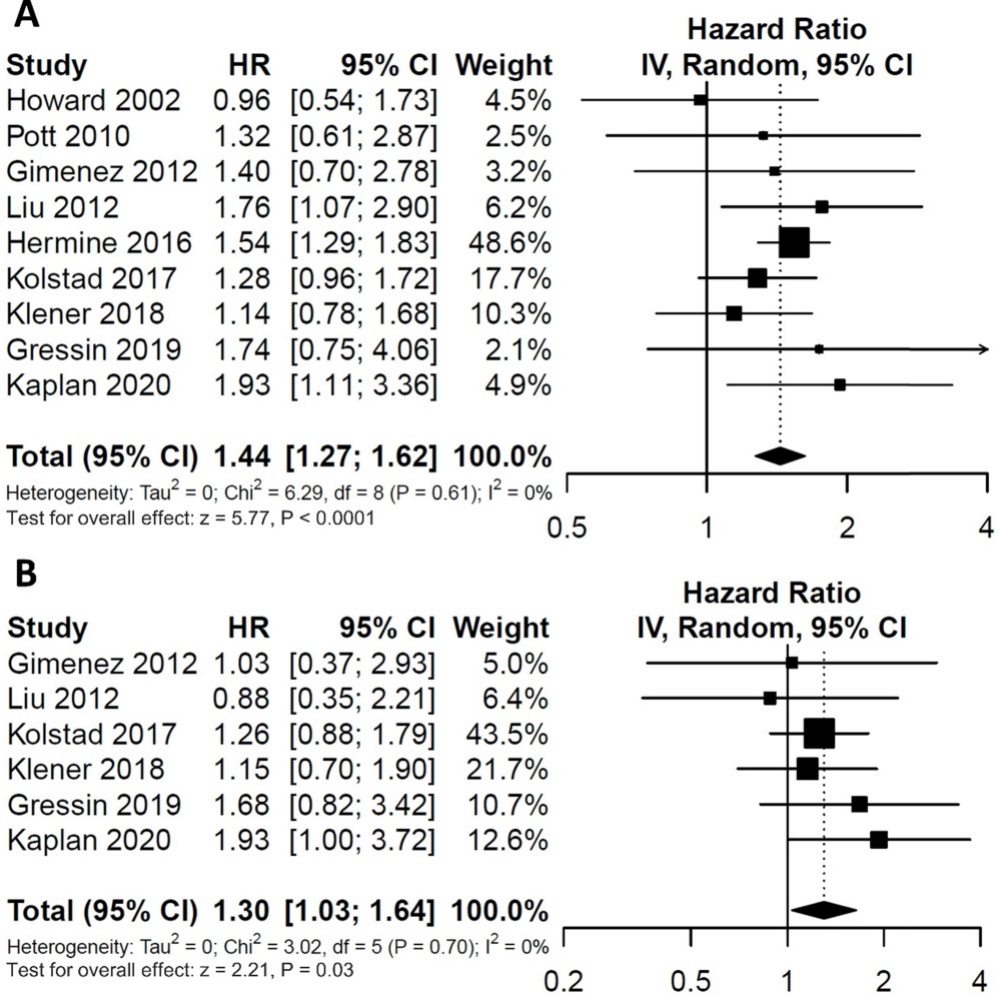
Forest plots for overall effect of post-induction minimal residual disease (MRD) status on (A) progression free survival (PFS) and (B) overall survival (OS).

**Figure 3:**
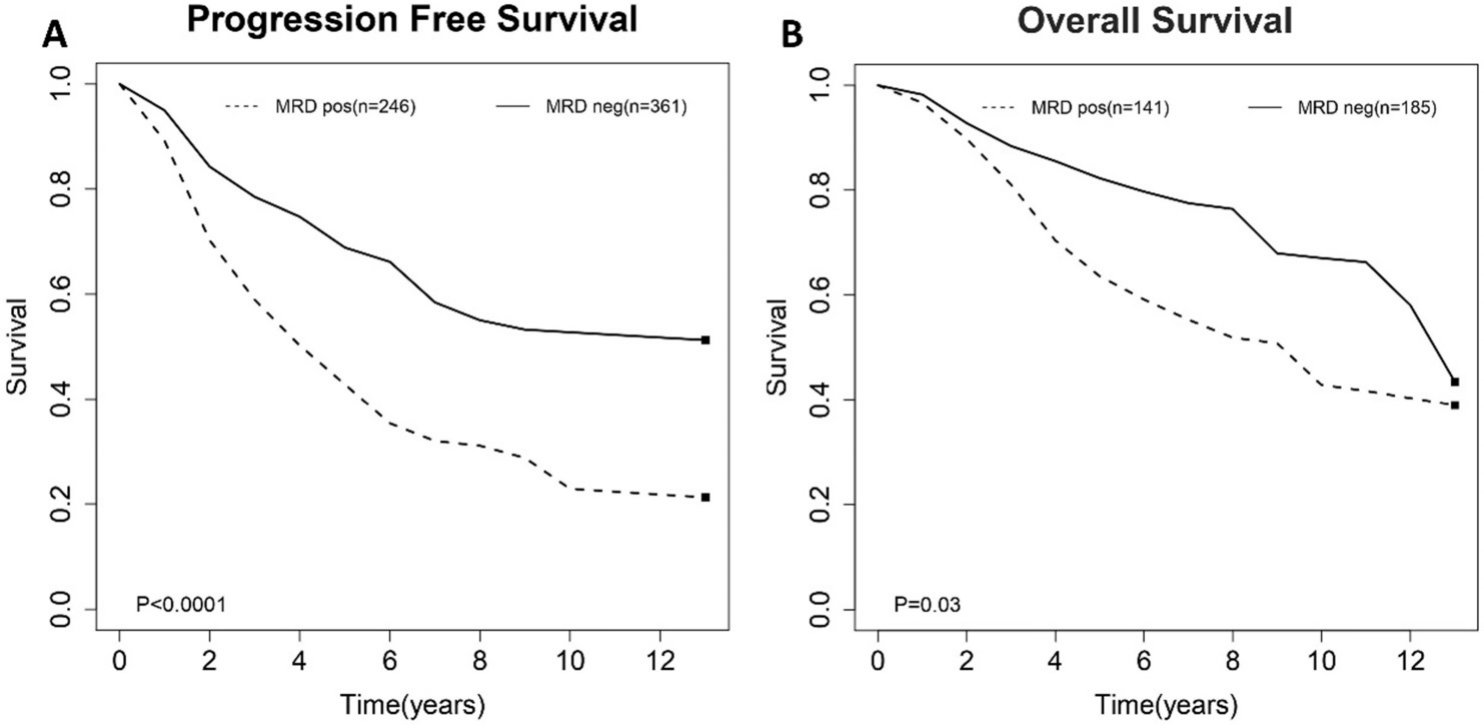
Estimated survival curves for (A) progression free survival (PFS) and (B) overall survival (OS) comparing post-induction minimal residual disease (MRD) positivity and negativity groups.

To evaluate the impact of post-induction MRD status on PFS, data were extracted from nine studies involving 607 patients (246 MRD-positive patients; 361 MRD-negative patients). For OS, data were extracted from six studies involving 326 patients (141 MRD-positive patients; 185 MRD-negative patients). Compared with MRD-negative patients, MRD-positive patients had shorter PFS (HR=1.44; 95%CI 1.27-1.62; *P*<0.0001; Figure 2A) and OS (HR=1.30; 95%CI 1.03-1.64; *P*=0.03; Figure 2B). The pooled 5-year PFS for MRD-positive patients and MRD-negative patients were 42.8% (95%CI 31.8%-57.6%) and 68.9% (95%CI 61.4%-77.3%) (Figure 3A). The pooled 5-year OS for MRD-positive patients and MRD-negative patients were 63.6% (95%CI 54.8%-73.8%) and 82.3% (95%CI 76.1%-88.9%) (Figure 3B). In the tests of heterogeneity, there were no significant differences among the included studies for PFS (χ^2^ =6.29, df=8; *P*=0.61; I^2^=0%) and OS (χ^2^ =3.02, df=5; *P*=0.70; I^2^=0%).

### Meta-analysis of post-consolidation MRD status

**Figure 4:**
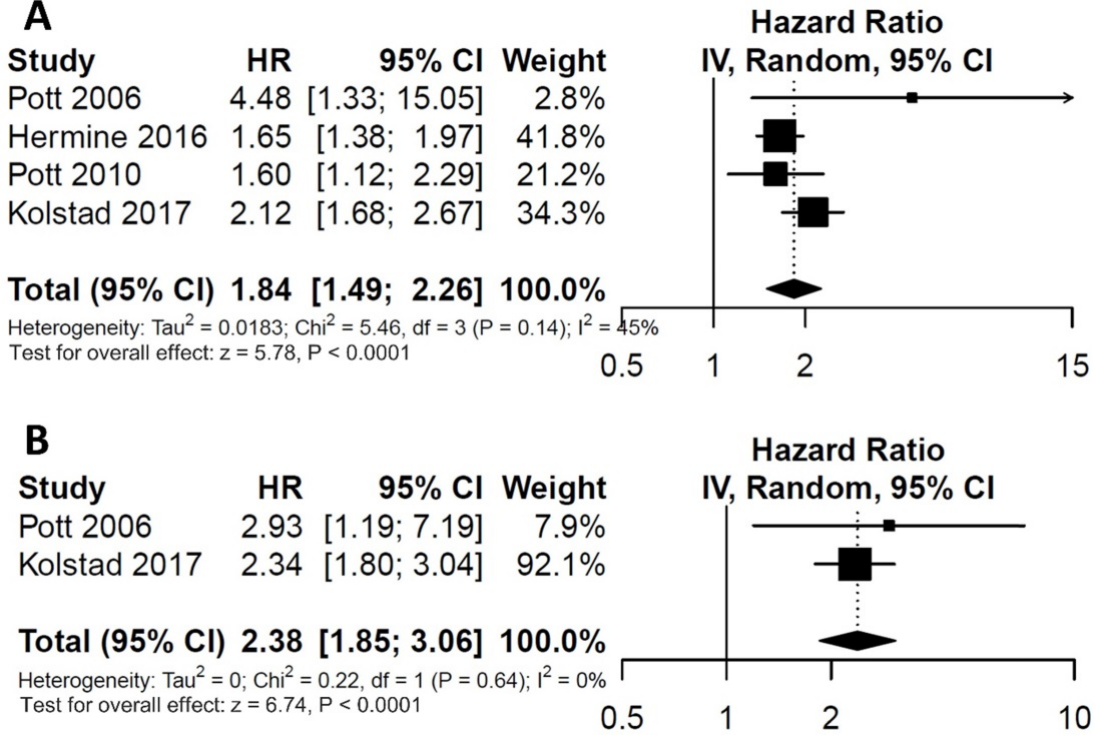
Forest plots for overall effect of post-consolidation minimal residual disease (MRD) status on (A) progression free survival (PFS) and (B) overall survival (OS).

**Figure 5:**
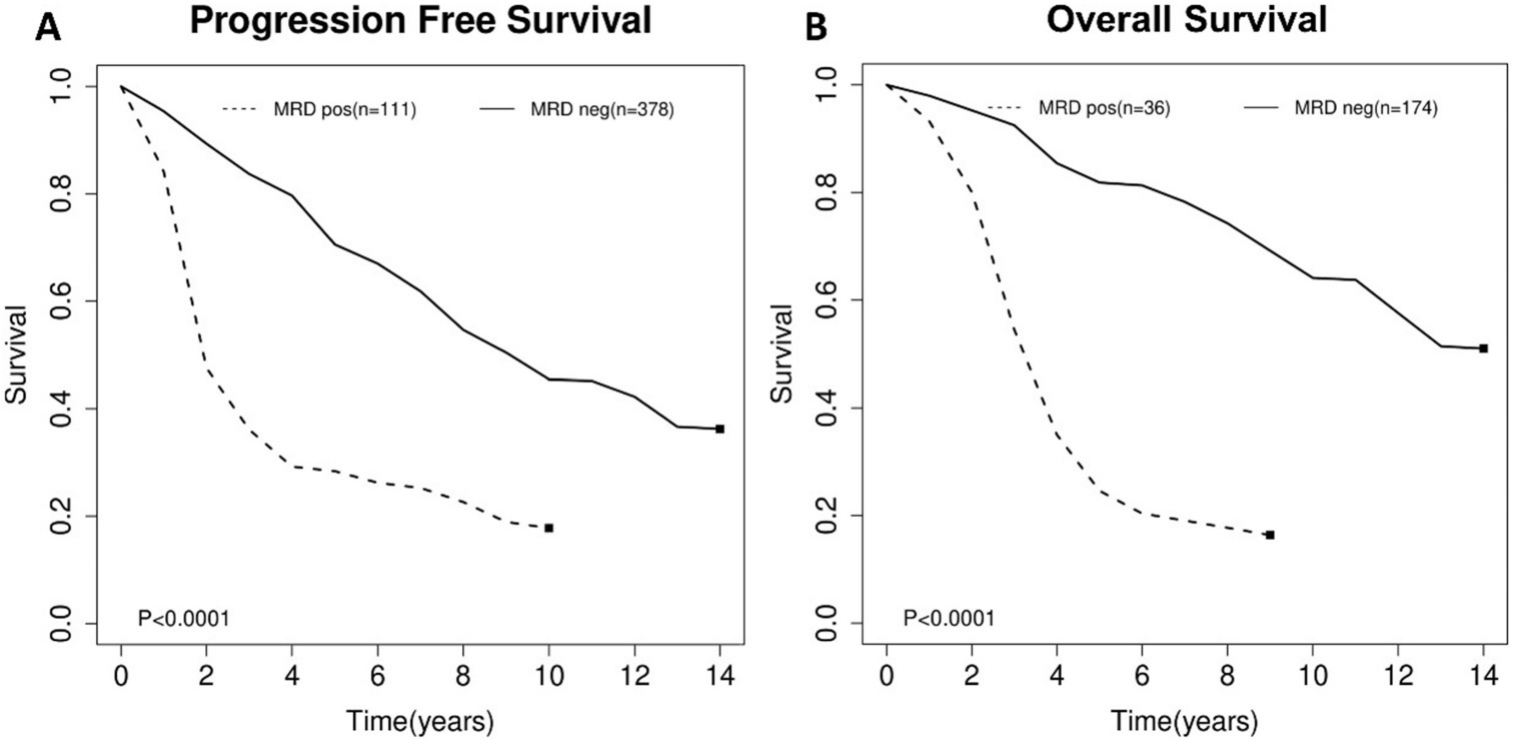
Estimated survival curves for (A) progression free survival (PFS) and (B) overall survival (OS) comparing post-consolidation minimal residual disease (MRD) positivity and negativity groups.

For the impact of post-consolidation MRD status on PFS, data from four studies involving 489 patients (111 MRD-positive patients; 378 MRD-negative patients) were extracted and analyzed. Concerning OS, data from 2 studies involving 210 patients (36 MRD-positive patients; 174 MRD-negative patients) were extracted. MRD positivity was associated with worse PFS (HR=1.84; 95%CI 1.49-2.26; *P*<0.0001; Figure 4A) than MRD negativity, with a moderate heterogeneity among the four studies (χ^2^ =5.46, cdf=3; *P*=0.14; I^2^=45%). The pooled 5-year PFS for MRD-positive patients and MRD-negative patients were 70.5% (95%CI 65.5%-76.0%) and 28.4% (95%CI 17.5-46.0%) (Figure 5A). As there was a moderated heterogeneity among the studies for PFS, we performed a sensitivity analysis by removing one trial each time to explore the source of heterogeneity. By removing the trial by Kolstad et al., the heterogeneity of the studies for PFS reduced (*P*=0.27; I^2^=24%). Patients with MRD positive status still tended to have shorter PFS (HR=1.67; 95%CI 1.43-1.95; *P*<0.0001).

As for OS, compared with MRD negativity, MRD positivity was significantly associated with shorter OS (HR=2.38; 95%CI 1.85-3.06; *P*<0.0001; Figure 4B). There was no significant difference among studies for OS in the test of heterogeneity (χ^2^ =0.22, df=1; *P*=0.64; I^2^=0%). The pooled 5-year OS for MRD-positive patients and MRD-negative patients were 24.6% (95%CI 11.9%-51.0%) and 81.1% (95%CI 75.5%-88.6%) (Figure 5B).

### Publication bias

Funnel plots indicated no significant publication bias (Supplementary Figure S1A, 2A, 3A & 4). In Begg's and Egger's tests, results showed no evidence of publication bias for the studies of post-induction MRD status for PFS (Begg's test: *P*=0.9195; Egger's test: *P*=0.7388), post-induction MRD status for OS (Begg's test: *P*=0.7500; Egger's test: *P*=0.2111) and post-consolidation MRD for PFS (Begg's test: *P*=0.7194; Egger's test: *P*=0.9701) (supplementary Table S3). Considering the relatively small number of included studies in our meta-analysis (n=10), small trial bias might exist. We performed trim and fill methods to adjust publication bias and regenerated funnel plots based on the adjusted results. The adjusted plots were showed in supplementary (Figure S1B, 2B and 3B). Of note, due to the limited number of included studies reported post-consolidation MRD status on overall survival (n=2), Begg's, Egger's test and trim and fill methods were not applicable for testing and adjusting publication bias of these two studies.

### Sensitivity analysis

We performed sensitivity analysis by removing one study each time. The forest plots were showed that the pooled results were not significantly changed by any single study. Results were illustrated in supplementary Figure S5, 6, 7 and 8.

## Discussion

In this meta-analysis, we performed quantitative synthesis to explore the association of MRD status on survival outcomes for newly diagnosed MCL. Results revealed that patients with MRD positive status had worse PFS and OS than patients with MRD negative status.

MRD status after both induction therapy and consolidation therapy showed prognostic value. This may provide information for deciding timing of MRD assessment. On the one hand, post-induction and post-consolidation MRD status assessment could be useful because it has prognostic value and may provide information for further therapy decision-making. There are some previous studies showed that rituximab consolidation post-ASCT could eliminate MRD and improve survival 17, 18, 32, 33.Therefore, surveillance of MRD status after ASCT can be helpful to discriminate patients with probable worse outcome and these patients can get survival benefits from further consolidation. On the other hand, as MRD implies the depth of molecular remission, continuous assessment of MRD status can be a useful tool for monitoring early relapse. At present, computed tomography (CT) and positron emission tomography (PET)/CT are the regular tools for assessing remission status, monitoring relapse and driving clinical treatment decisions. Our study results indicated that for MCL patients, MRD status at certain time had strong predictive power for relapse and death during the whole treatment procedure. Therefore, MRD assessment may be a supplement for the regular imaging response monitoring in the future. Although MRD status is of prognostic value based on this meta-analysis, the recommendation of clinical application of MRD, especially MRD-driven treatment decision, still needs to be validated in large prospective cohort study. In fact, there is an ongoing prospective randomized, phase Ⅲ study focusing on the impact of treatment options on survival outcomes in MRD negative MCL patients (NCT03267433). In this study, MCL patients in MRD-negative first remission will be randomized to undergo autologous hematopoietic stem cell transplantation (auto-HCT) followed by rituximab maintenance group or rituximab maintenance alone (without auto-HCT). Survival results of patients who are MRD-negative and patients who are MRD-positive or MRD-indeterminate prior to auto-HCT will be explored. This study will provide more information on the prognostic value of MRD status in MCL patients and the clinical efficacy of consolidation and maintenance treatment for patients with different post-induction MRD status.

The induction treatments varied across the included studies. For the analysis of post-induction MRD status, the consolidation and maintenance protocols also varied. Patients in the five included studies received ASCT while patient in the other four studies did not receive ASCT. Also, patients of four studies received maintenance treatment after induction or consolidation. Despite of the differences, all the studies showed a consistent effect of MRD on PFS and OS, confirmed by non-significant heterogeneity in Cochrane *Q* test and *I*^2^ statistic tests. This indicated that overall effect of MRD status did not be influenced by treatment. MRD can be a prognostic marker independent of the induction, consolidation, and maintenance treatments. However, the probability that negative results tend less likely to be reported always exists and this may cause inevitable bias in meta-analysis. We suggest that future clinical trials and real-world practice are necessary to further assess this question.

Only one of included study reported MRD status and survival outcomes in patients achieved conventional complete remission (CR). Due to limited data, we did not pool the results to assess the prognostic value of MRD status in clinical CR patients' subgroup. But some previous studies showed that MRD negativity was emerging as a significant therapeutic goal instead of clinical CR. A study based on the randomized intergroup trials of the EU-MCL Network showed that among 406 patients in remission 6 months after ASCT or end of induction, a positive MRD status in PB was highly associated with a shorter PFS and OS 34. Seventy-six MRD-positive patients confirmed clinical relapse, with only 10 patients being MRD-negative. Another retrospective study included MCL patients who underwent ASCT in clinical CR between 1996 and 2011 35. Of 75 patients achieving CR, 11% (8/75) were MRD-positive. MRD-positive was associated with worse PFS (adjusted HR 4.043; 95%CI 1.429-11.442; *P*=0.0085) and OS (adjusted HR 3.68; 95%CI 1.55-8.79; *P*=0.0033). These results indicate that instead of conventional CR, MRD can be an important endpoint in clinical trials.

We identified two conference abstracts that could not be included into this meta-analysis due to insufficient data and lack of peer-review. One abstract by Torka et al. explored prognostic value of MRD in a single-arm, open-label, multi-center phase Ⅱ study 36. 37 patients received O-HyperCVAD/MA (ofatumumab, cyclophosphamide, vincristine, doxorubicin, and dexamethasone alternating with high-dose methotrexate and cytarabine) as induction treatment in this study. ASCT and post-ASCT rituximab maintenance were performed. MRD status was assessed in 28 of 37 patients. Results showed that MRD negativity after 2 cycles of induction treatment was associated with improved PFS (*P*=0.04) and OS (*P*=0.03). Another abstract by Smith et al. reported the results of ECOG1411 randomized phase II trial 37. Patients were randomly assigned to receive BR (bendamustine and rituximab) with or without bortezomib as induction treatment, followed by rituximab with or without lenalidomide as consolidation treatment. MRD status after 3 cycles of induction treatment was assessed in 189 patients. Results showed that mid-induction MRD status was correlated with PFS (*P* <0.01). One of the included studies in our meta-analysis also reported the association of mid-induction MRD status and survival outcomes 30. However, in this study, MRD status had no significant relevance with PFS and OS. These results showed that early MRD remission might predict survival. But the utility of mid-induction MRD status still needs to be confirmed in future trials.

In this study, we did not analyze the survival results of different detection methods as all the included studies adopted PCR as detection method (Table 1). Currently, multiparameter flow cytometry (MFC) and real-time quantitative polymerase chain reaction (RQ-PCR) are both the methods of choice for MRD detection. MFC is based on immunophenotype and can be well performed in the diagnosis of lymphoma at short detection time and relatively low cost. However, compared with PCR, the lower sensitivity of MFC limited its use in the follow-up period 38, 39. Based on IGH rearrangement or t(11;14), RQ-PCR is a sensitive tool for detecting MRD in follow-up period and its sensitivity can reach up to 10^-5^ 12, 40. But for patients without canonical translocations, the allele-specific oligonucleotide (ASO) primer design is necessary and this time-consuming procedure limited the applicability. Recently, the advent of next generation sequencing (NGS) technique provides another powerful tool for MRD detection. This is a well-developed technique and has been applied in recent clinical trials of MCL 37. Results showed that NGS could be more sensitive than FC when detection material was available. Based on the results mentioned above, we suggest that when applying MRD assessment in clinical practice, the methods for MRD detection should be determined to specific circumstances and should follow standardized protocols.

There are limitations of our meta-analysis that should be considered. Analysis according to other variables, such as MRD cut-off value and type of induction therapies, were not performed due to the small number of studies in each subgroup. Also, as we could not get access to individual-level data, the pooled survival rates at certain time points were estimated and we could not draw strong conclusion of the survival probabilities.

## Conclusion

In summary, results of this meta-analysis showed that MRD positivity after induction and consolidation treatments was associated with worse PFS and OS for MCL. MRD-based treatment strategies should be further explored in clinical trials and real-world practice.

## Supplementary Material

Supplementary figures and tables.

## Figures and Tables

**Table 1:** Characteristics of included studies

Study	Region	MRD+ pts number	MRD- pts number	MRD source	MRD detection method	MRD cut-off value	Median follow-up (months)	Median age (years)	MRD related outcomes	Induction	ASCT	Maintenance
Howard 2002	North America	16	9	PB, BM	PCR	10^-5^	25.0	55	PFS	R-CHOP	No	None
Pott 2006	Europe	13	14	PB, BM	PCR	10^-4^	48.0	56	PFS, OS	COP, CHOP, PmM	Yes	None
Pott 2010^†^	Europe	69^‡^	87^‡^	PB, BM	PCR, FC	10^-4^	17.0	61	PFS	R-CHOP, R-FC	No	Interferon-α or rituximab
		20^§^	31^§^									
Gimenez 2012	Europe	8	9	PB, BM	PCR	10^-5^	NR	55	PFS, OS	(R)VAD+C	Yes	None
Liu 2012	North America	21	18	PB, BM	PCR	10^-4^ to 10^-5^	NR	56	PFS, OS	RM-CHOP	Yes	Rituximab
Hermine 2016	Europe	65^a^	126^a^	PB, BM	PCR	10^-4^	73.2	55	PFS	Alternating R-CHOP/R-DHAP or R-CHOP	Yes	None
		55^b^	173^b^									
Kolstad 2017	Europe	54^a^	76^a^	PB, BM	PCR	10^-4^ to 10^-6^	102.0	57	PFS, OS	Alternating Maxi-CHOP-R/R-Ara-C	Yes	None
		23^b^	160^b^									
Klener 2018	South America	22	30	PB, BM	PCR	10^-4^	54.0	70	PFS, OS	Alternation R‐CHOP/R-Ara-C	No	Rituximab
Gressin 2019	Europe	11	35	PB, BM	PCR	10^-5^	52.0	73	PFS, OS	RiBVD	No	None
Kaplan 2020	North America	25	17	BM	PCR	10^-4^ to 10^-5^	99.6	59	PFS, OS	RM-CHOP	Yes	Bortezomib

^†^ Only data of MCL Elderly study was included in analysis. ^‡^ Patients number after induction treatment. ^§^ Patients number after ASCT or during first 12 months of maintenance treatment. *Abbreviations*: pts, patients; MRD+, minimum residual disease positive; MRD-, minimum residual disease negative; PB, peripheral blood; BM, bone marrow; PCR, polymerase chain reaction; R-CHOP, rituximab, cyclophosphamide, doxorubicin, vincristine and prednisone; COP, cyclophosphamide, vincristine and prednisone; CHOP, cyclophosphamide, doxorubicin, vincristine and prednisone; PmM, prednimustine, mitoxantrone; ASCT, autologous stem cell transplantation; FC, flow cytometry; R-DHAP, rituximab with high-dose cytarabine and cisplatin; R-FC, rituximab, fludarabine and cyclophosphamide; NR, not reported; (R)VAD+C, (rituximab) vincristine, doxorubicin, dexamethasone + chlorambucil; RM-CHOP, rituximab, methotrexate, cyclophosphamide, doxorubicin, vincristine, and prednisone; Maxi-CHOP-R, cyclophosphamide, doxorubicine, prednisolone, rituximab; R-Ara-C, rituximab, cytarabine; RiBVD, rituximab, bortezomib, bendamustine and dexamethasone.

## Abbreviations

MCL: mantle cell lymphoma
NHL: non-Hodgkin lymphoma
ASCT: autologous stem cell transplantation
MRD: minimal residual disease
PB: peripheral blood
BM: bone marrow
PFS: progression free survival
OS: overall survival
HR: hazard ratio
CI: confidence interval
PCR: polymerase chain reaction
FC: flow cytometry
CR: complete remission
