# Supplementary material for: Minimal Residual Disease and Survival Outcomes in Patients with Mantle Cell Lymphoma: a systematic review and meta-analysis
Source: J Cancer. 2021 Jan 1;12(2):553–61. doi: 10.7150/jca.51959 (PMC7738989; doi:10.7150/jca.51959)

Table S1. Search strategies.

PUBMED	LINE	SEARCH TERM
	1	((((((((neoplasm, residual[MeSH Terms]) OR (neoplasm, residual[Title/Abstract])) OR (Minimal Residual Disease*[Title/Abstract])) OR (Residual Disease, Minimal[Title/Abstract])) OR (Residual Minimal Disease*[Title/Abstract])) OR (Residual Tumor*[Title/Abstract])) OR (Residual Cancer*[Title/Abstract])) OR (Tumour, Residual[Title/Abstract])) OR (Cancer, Residual[Title/Abstract])
	2	((((((Lymphoma, Mantle-Cell[MeSH Terms]) OR (Lymphoma, Mantle-Cell[Title/Abstract])) OR (Lymphoma, Mantle Cell[Title/Abstract])) OR (Lymphomas, Mantle-Cell[Title/Abstract])) OR (Mantle-Cell Lymphoma[Title/Abstract])) OR (Mantle Cell Lymphoma[Title/Abstract])) OR (MCL[Title/Abstract])
	3	1 AND 2
**EMBASE**	**LINE**	**SEARCH TERM**
	1	'lymphoma, mantle-cell'/exp
	2	'lymphoma, mantle-cell':ab,ti OR 'lymphoma, mantle cell':ab,ti OR 'lymphomas, mantle-cell':ab,ti OR 'mantle-cell lymphoma':ab,ti OR 'mantle cell lymphoma':ab,ti OR mcl:ab,ti
	3	#1 OR #2
	4	'neoplasm, residual'/exp
	5	'neoplasm, residual':ab,ti OR 'minimal residual disease*':ab,ti OR 'residual disease, minimal':ab,ti OR 'residual minimal disease*':ab,ti OR 'residual tumor*':ab,ti OR 'residual cancer*':ab,ti OR 'tumour, residual':ab,ti OR 'cancer, residual':ab,ti
	6	#4 OR #5
	7	#3 AND #6
	8	'meta' OR 'systematic analysis' OR 'review'/exp
	9	'animal'/exp NOT 'human'/exp
	10	'clinical trial':ab,ti OR 'randomized clinical trial':ab,ti OR 'prospective study':ab,ti OR 'prospective trial':ab,ti OR 'clinical study':ab,ti OR 'cohort study':ab,ti
	11	#7 NOT #8

	12	#11 NOT #9
**WEB OF SCIENCE**	**LINE**	**SEARCH TERM**
	1	TS=('Lymphoma, Mantle-Cell' OR 'Lymphoma, Mantle Cell' OR 'Lymphomas, Mantle-Cell' OR 'Mantle-Cell Lymphoma' OR 'Mantle Cell Lymphoma' OR 'MCL')
	2	TI=('Lymphoma, Mantle-Cell' OR 'Lymphoma, Mantle Cell' OR 'Lymphomas, Mantle-Cell' OR 'Mantle-Cell Lymphoma' OR 'Mantle Cell Lymphoma' OR 'MCL')
	3	AB=('Lymphoma, Mantle-Cell' OR 'Lymphoma, Mantle Cell' OR 'Lymphomas, Mantle-Cell' OR 'Mantle-Cell Lymphoma' OR 'Mantle Cell Lymphoma' OR 'MCL')
	4	#1 OR #2 OR #3
	5	TS=('neoplasm, residual' OR 'Minimal Residual Disease*' OR 'Residual Disease, Minimal' OR 'Residual Minimal Disease*' OR 'Residual Tumor*' OR 'Residual Cancer*' OR 'Tumor, Residual' OR 'Cancer, Residual')
	6	TI=('neoplasm, residual' OR 'Minimal Residual Disease*' OR 'Residual Disease, Minimal' OR 'Residual Minimal Disease*' OR 'Residual Tumor*' OR 'Residual Cancer*' OR 'Tumor, Residual' OR 'Cancer, Residual')
	7	AB=('neoplasm, residual' OR 'Minimal Residual Disease*' OR 'Residual Disease, Minimal' OR 'Residual Minimal Disease*' OR 'Residual Tumor*' OR 'Residual Cancer*' OR 'Tumor, Residual' OR 'Cancer, Residual')
	8	#5 OR #6 OR #7
	9	#4 AND #8
	10	TS=(animal* NOT human*)
	11	TS=(meta OR systematic analysis OR review)
	12	#9 NOT #10
	13	#12 NOT #11
**THE COCHR**	**LINE**	**SEARCH TERM**
	1	Lymphoma, Mantle-Cell in Trials

**ANE LIBRARY**	2	(mantle cell lymphoma):ti,ab,kw OR (MCL):ti,ab,kw OR (lymphoma, mantle cell):ti,ab,kw
	3	1 OR 2
	4	(neoplasm, residual):ti,ab,kw
	5	(Minimal Residual Disease*):ti,ab,kw OR (Residual Disease, Minimal):ti,ab,kw OR (Residual Minimal Disease*):ti,ab,kw OR (Residual Tumor*):ti,ab,kw OR (Residual Cancer*):ti,ab,kw OR (Tumor, Residual):ti,ab,kw OR (Cancer, Residual):ti,ab,kw
	6	#3 AND #5

Table S2. Quality assessment of included studies using Newcastle-Ottawa quality assessment scale.

Study	Selection				Comparability	Outcome			Total
	Representativeness of exposed cohort	Selection of the non-exposed cohort	Ascertainment of exposure to implants	Demonstration that outcome of interest was not present at start of study	Comparability of cohorts based on the design or analysis	Assessment of outcome	Follow-up long enough for outcomes to occur	Adequacy of follow up of cohorts	
Howard 2002	1	1	1	1	0	1	1	1	7
Pott 2006	1	1	1	1	2	1	1	1	9
Pott 2010	1	1	1	1	0	1	1	1	7
Gimenez 2012	1	1	1	1	0	1	1	1	7
Liu 2012	1	1	1	1	0	1	1	1	7
Hermine 2016	1	1	1	1	2	1	1	1	9
Kolstad 2017	1	1	1	1	0	1	1	1	7
Klener 2018	1	1	1	1	0	1	1	1	7
Gressin 2019	1	1	1	1	0	1	1	1	7
Kaplan 2020	1	1	1	1	0	1	1	1	7

Table S3. Publication bias test.

**Study group**	**P value (Begg's test)**	**P value (Egger's test)**
**Progression free survival**		
Studies of post-induction MRD	0.9195	0.7388
Studies of post-consolidation MRD	0.7500	0.2111
**Overall survival**		
Studies of post-induction MRD	0.7194	0.9701
Studies of post-consolidation MRD	Not applicable*	Not applicable*

*Due to the limited number of included studies reported post-consolidation MRD status on overall survival (n=2), Begg's and Egger's test was not applicable.

Figure S1. Funnel plot of progression-free survival for studies reported post-induction MRD status. (A) Before adjustment; (B) After adjustment.

(A)

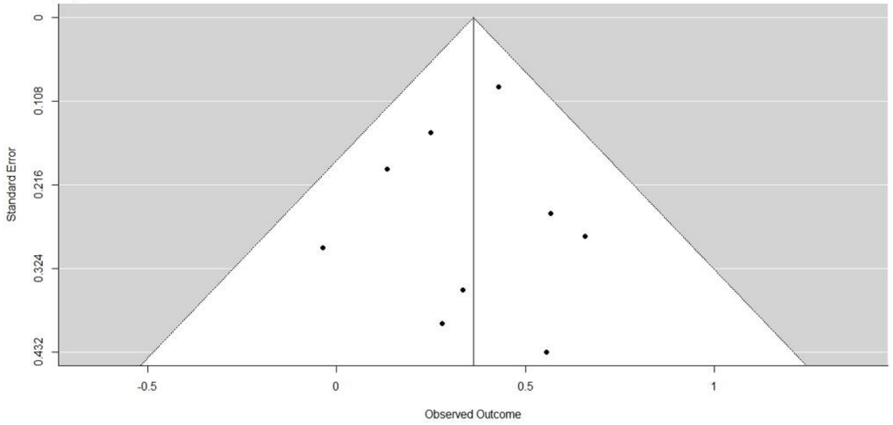

(B)

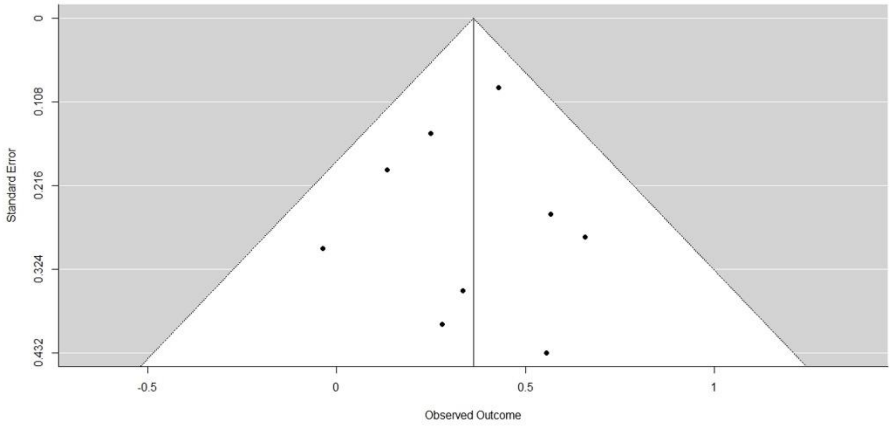

Figure S2. Funnel plot of overall survival for studies reported post-induction MRD status. (A) Before adjustment; (B) After adjustment.

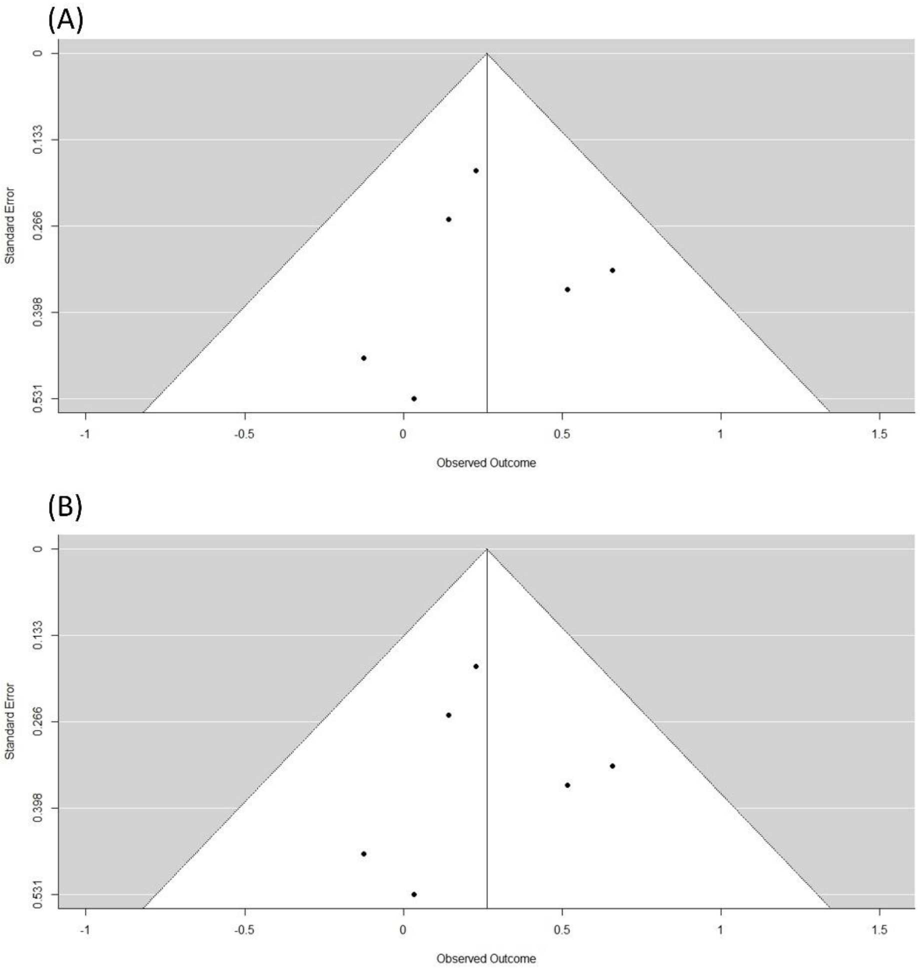

Figure S3. Funnel plot of progression free survival for studies reported post-consolidation MRD status. (A) Before adjustment; (B) After adjustment.

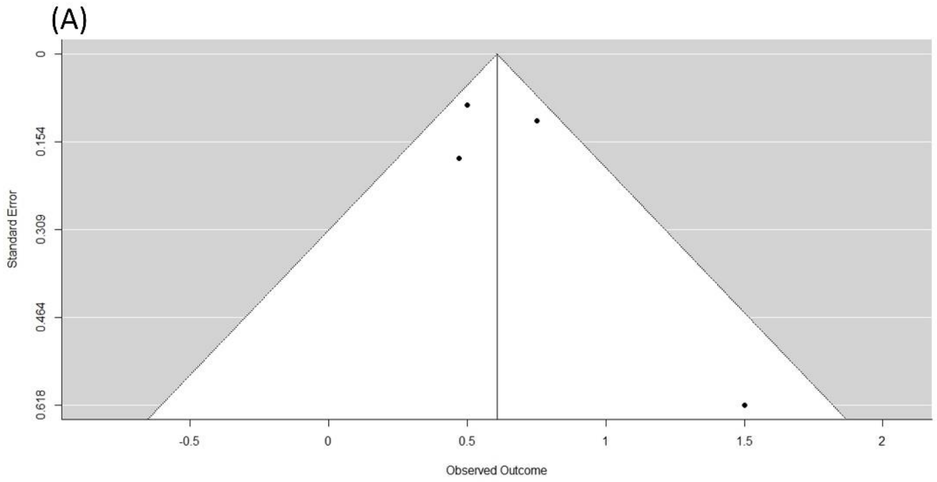

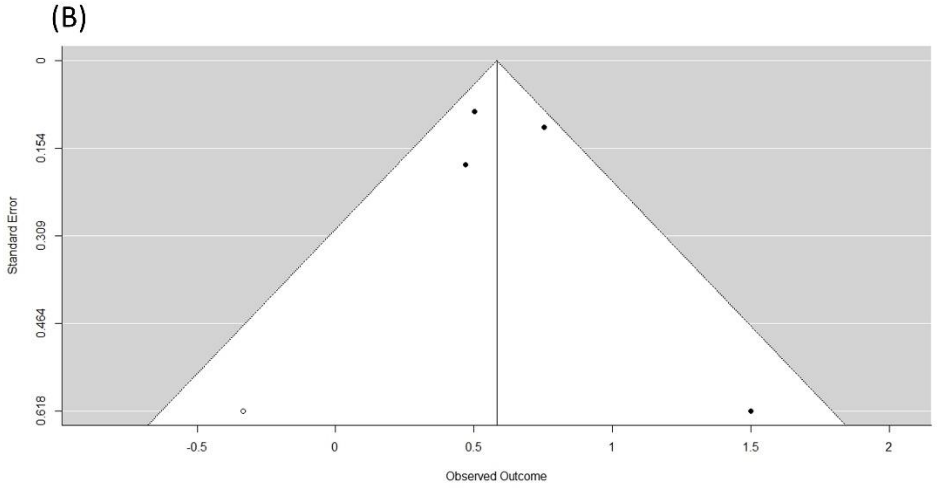

Figure S4. Funnel plot of overall survival for studies reported post-consolidation MRD status.

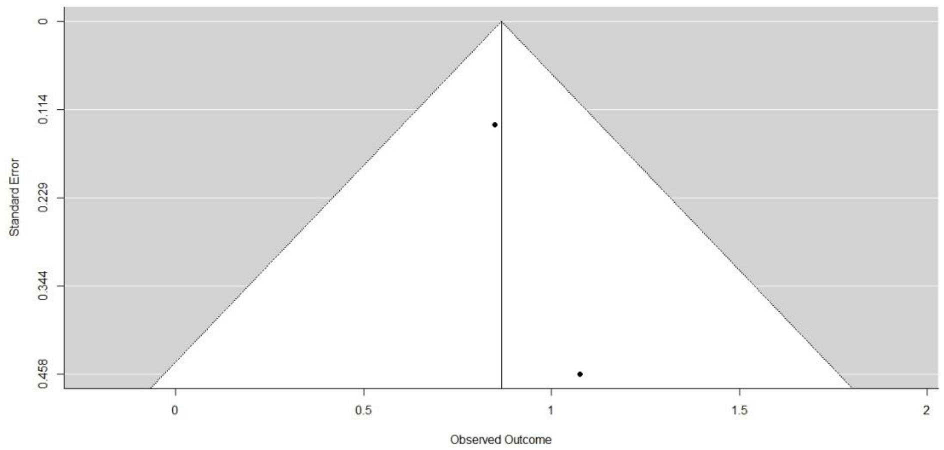

Figure S5. Sensitivity analysis of progression free survival for studies reported post-induction MRD status.

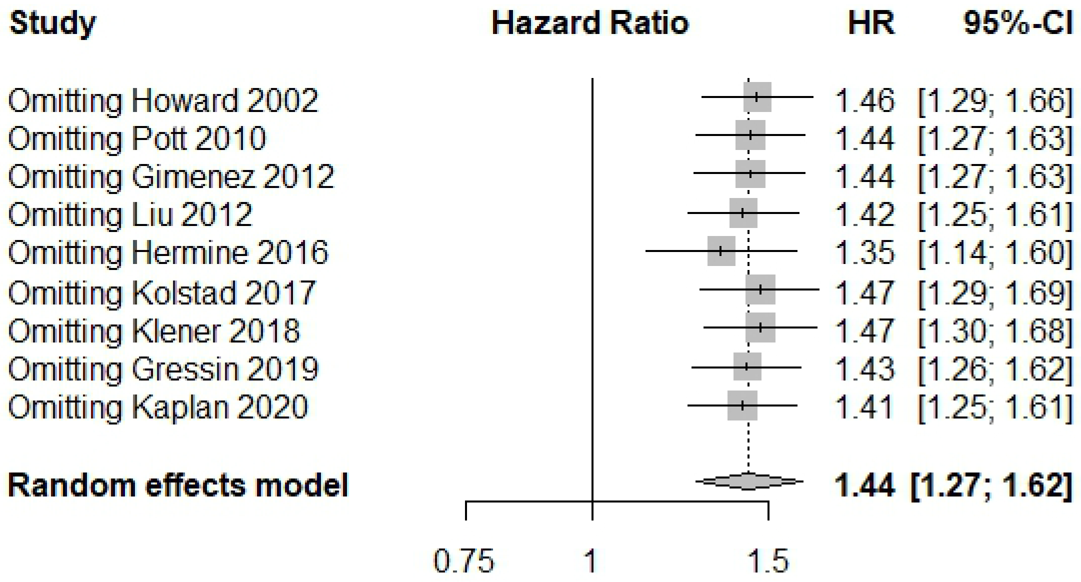

Figure S6. Sensitivity analysis of overall survival for studies reported post-induction MRD status.

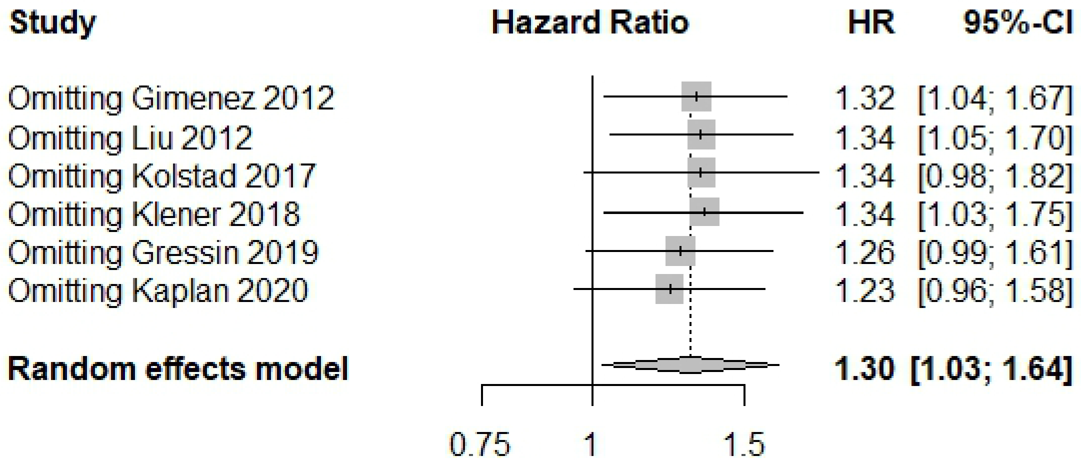

Figure S7. Sensitivity analysis of progression free survival for studies reported post-consolidation MRD status.

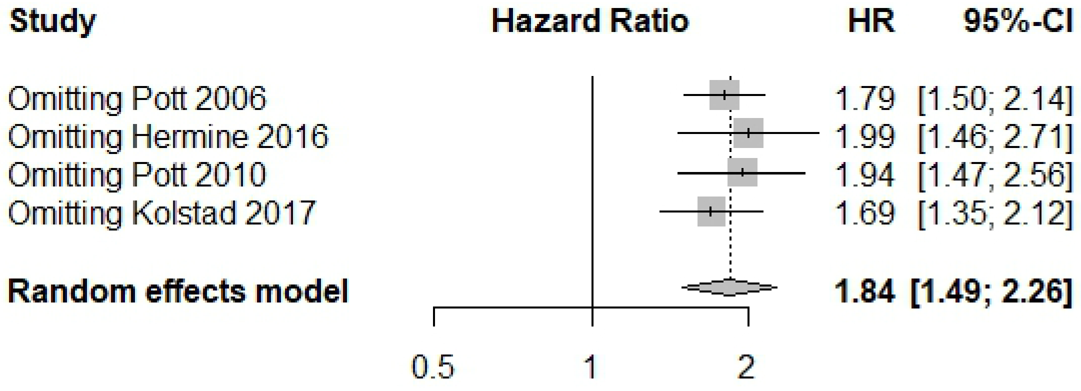

Figure S8. Sensitivity analysis of overall survival for studies reported post-consolidation MRD status.

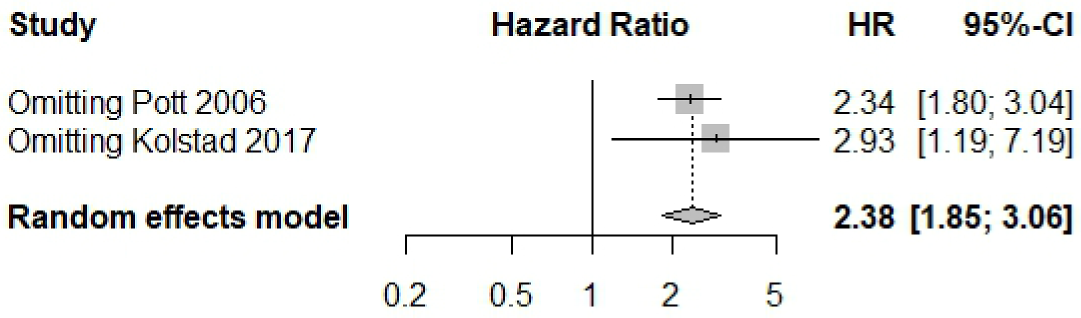

Supplement: Supplementary file 1 — Supplementary figures and tables. [file jcav12p0553s1.pdf]
